# Good syndrome presenting with CD8+ T-Cell large granular lymphocyte leukemia

**DOI:** 10.18632/oncotarget.5369

**Published:** 2015-09-29

**Authors:** Caroline Caperton, Sudhanshu Agrawal, Sudhir Gupta

**Affiliations:** ^1^ Program in Primary Immunodeficiency and Aging, Division of Basic and Clinical Immunology, University of California at Irvine, Irvine, California, USA

**Keywords:** IFNgamma, PD-1, ICOS, memory T, granzyme

## Abstract

Good Syndrome is an adult-onset combined immunodeficiency defined by hypogammaglobulinemia, low or absent number of B cells, T cell deficiency and thymic tumor. We have characterized CD8+ T cells from a patient with Good syndrome that presented with CD8+T-cell large granular lymphocytic leukemia (LGL). Characterization of peripheral blood CD8+ T cells revealed that majority of CD8+ T cells were terminally differentiated effector memory phenotype (T_EMRA_; CD8+CCR7-CD45RA+), and were PD-1^high^ (CD279), ICOS^low^ (CD278), and granzyme^high^. Almost all CD8+ T cells were IFN-γ+. CD8 Treg (CD8+CD183+CCR7+CD45RA-) were decreased. T_EMRA_ phenotype along with CD279^high^, demonstrates that these are exhausted CD8+ T cells. This phenotype along with CD278^low^ may also explain severe T cell functional deficiency in our patient. In the present patient, T-LGL appears to be a clonal expansion of CD279+granzyme+IFN-γ+CD8+T_EMRA_ cells. To best of our knowledge this is the first case of CD8+T-cell LGL leukemia associated with Good syndrome.

## INTRODUCTION

Good Syndrome is a rare adult-onset primary combined immunodeficiency characterized by hypogammag lobulinemia, reduced or absent B cells, T cell deficiency, and thymoma [1–3]. Patients usually present at mean age of 56 years, with males and females equally affected. There is an increased susceptibility to frequent infections with bacteria, viruses, fungi, and parasites [4–6]. In addition, there is an increased incidence of autoimmune diseases in Good syndrome, including red cell aplasia, myasthenia gravis, neutropenia, pemphigus, lichen planus, and inflammatory bowel diseases [6–8]. The majority of thymoma are benign; more than 50% are spindle cells type, and approximately 10% of thymoma are malignant. Few cases of monoclonal gammopathy of undetermined significance (MGUS) have been reported in Good syndrome [8, 9]. Malignancy in Good syndrome is exceedingly rare.

Large granular lymphocyte (LGL) leukemia is a group of rare clonal lymphoproliferative diseases. They can be of T lymphocytes or natural killer cell lineages. These diseases frequently present with neutropenia, and autoimmune diseases [10–12]. T-LGL leukemia (CD3+ CTL) is more commonly of a chronic and indolent nature; neutropenia is present in approximately 80% of cases, and severe neutropenia in 45% of cases. CD3-CD56+ NK cell LGL is highly aggressive, occurs in younger patients, and EBV has been linked to its pathogenesis [13]. The pathogenesis T-LGL is unclear; however, dysregulated activation signals, and impaired apoptosis have been suggested to its pathogenesis [14]. T- LGL has never been reported with Good syndrome, and CD8+ T cells have not been extensively characterized in T-LGL.

We report a case of an adult patient who initially presented with thymoma and T-cell large granular lymphocytic leukemia (LGL), and later was confirmed to have a combined immunodeficiency consistent with a diagnosis of Good syndrome. We present an extensive characterization of his CD8+ T cells that demonstrates that these cells have a phenotype of exhausted T cells, which may be responsible, in part, for severe immunodeficiency in our patient.

## RESULTS

### Patient

The patient is a previously healthy 58 year-old Asian male who was referred to one of us (SG) for immunological evaluation. Originally he presented with progressive neck pain, back pain, fatigue, unintentional weight loss of 10 pounds in one year, and chronic cough that began one year prior to presentation. Complete blood count found revealed severe macrocytic anemia with hemoglobin of 6 g/dL, requiring four blood transfusions. Chest radiograph revealed a mediastinal mass, which was excised, and pathology showed morphology compatible with a Type A thymoma of the current WHO classification of thymic tumors. Bone marrow biopsy at that time revealed only decreased erythropoiesis and he was treated with prednisone for a diagnosis of aplastic anemia. His clinical course was complicated by anemia requiring multiple blood transfusions, neutropenia requiring granulocyte-colony stimulating factor, opportunistic infections, including cytomegalovirus retinitis, and cutaneous fungal infections. Family history was significant for mother, maternal aunt, and sister all deceased from gastric cancer. Sister was diagnosed with BRCA1 positive ovarian cancer, and a brother with squamous cell carcinoma of the tongue.

### Diagnosis of T cell LGL

**Figure 1:**
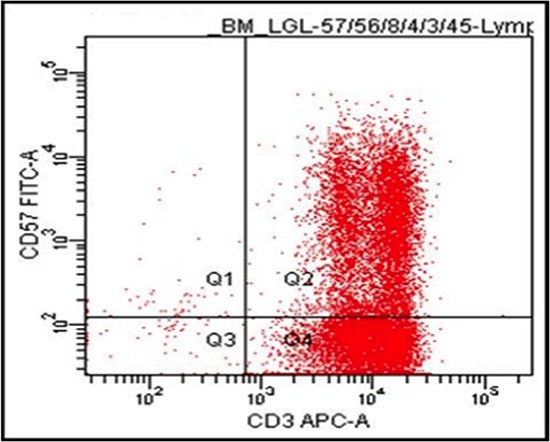
Flow cytometry on bone marrow cells Majority of cells are CD3+CD57+, a feature of T-LGL.

Repeat bone marrow aspiration confirmed a diagnosis of T cell large granulocyte leukemia (LGL) by flow cytometry initially as CD3+CD57+ (Figure 1) and then by more extensive phenotypic analysis as CD2+CD3+CD5dimCD7+CD8+CD57+CD56-TCRisά/β and by PCR for clonality. The LGLs comprised approximately 42% of nucleated cells and 68% of lymphocytes. Clonal rearrangements of both TCRβ and γ chains were detected by PCR, consistent with the diagnosis of T-cell LGL leukemia. T cell clonality screening by TCRγ PCR was positive for a clonal TCRβ gene rearrangement [15]. Results showed an oligoclonal pattern, with three or more distinct peaks present that met the criteria for clonality compared to the polyclonal background. The overall findings were consistent with bone marrow involvement by T-cell large granular lymphoproliferative disorder with molecular evidence of T-cell clonality. There was no immunophenotypic evidence of paroxysmal nocturnal hemoglobinuria by flow cytometry (data not shown). FISH analysis utilizing probes specific for aberrations commonly associated with myelodysplasia (MDS) and for rearrangements of the TCR alpha/delta locus (14q11) were performed. Cytogenetic analysis by FISH revealed no chromosomal abnormality in 200–300 nuclei/probe examined. FISH study did not detect aberrations MDS.

### Diagnosis of good syndrome

Table 1 shows Immunological analysis of the patient, which revealed decreased serum IgG, IgM, and IgA, markedly increased proportions and numbers of CD3+CD8+ T cells, and decreased proportions and numbers of CD3-CD16+CD56+ NK cells, CD3+CD4+ T helper cells, and absence of CD19+ B cells. Specific antibody responses to pneumococcus polysaccharides following Pneumovax-23 were lacking. The proliferative response to mitogens (PHA, Con A, PWM) and recall antigens (Candida, tetanus toxoid, mumps) were impaired. This combined immunodeficiency associated with thymoma established a diagnosis of Good syndrome. Neutrophil function (oxidative burst) was normal; however, a modest impairment of phagocytosis was observed.

**Table 1:** Patient's Immunological analysis

Tests	Patient	Controls (ranges)
**Serum Immunoglobulins (mg/dl)**		
IgG	381	700–1600
IgM	<25 mg	40–230
IgA	63	70–400
**Lymphocyte subsets % (absolute #)**		
CD3+	97 (2328)	61–88 (896–2210)
CD3+CD4+	24 (576)	33–63 (420–1248)
CD3+CD8+	69 (1656)	17–39 (255–1014)
CD4/CD8 ratio	0.35	1.03–3.24
CD3-CD16+CD56+	2 (48)	5–24 (120–513)
CD3-CD19+	0 (0)	7–22 (98–440)
**Anti-pneumococcal antibodies**	No response	19/23–22/23 serotype+
**Lymphocyte Proliferation (cpm)**		
PHA	81,691	101,501–328,377
Con A	64,860	138,643–312,665
PWM	18,198	32,087–88,020
Candida	6,748	13,478–164,075
Tetanus Toxoid	0	10,892–60,819
Mumps	0	3,423–22,333
PPD	738	0–11,914
**Neutrophil Functions**		
Oxidative burst (DHR, MFC#)	249	50–157
Phagocytosis (%)	64	95–100

### Characterization of peripheral blood CD8+ T cells

**Figure 2:**
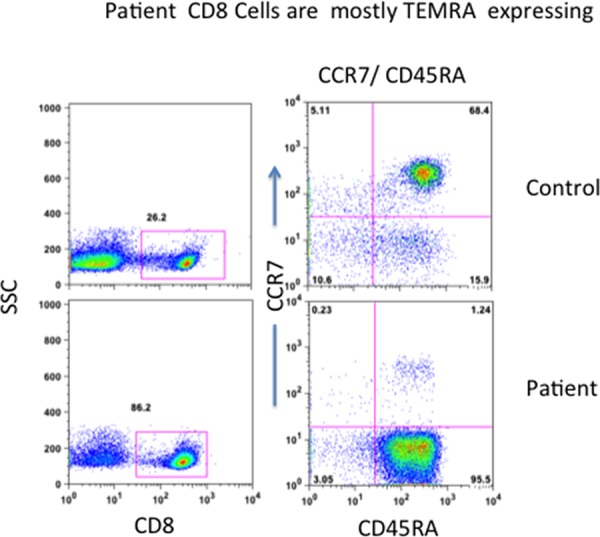
Peripheral blood T_N_, T_CM_, T_EM_, and T_EMRA_ CD8+ T cells Cells were stained with anti-CD8, anti-CCR7, and anti-CD45RA and isotype controls. CD8+ T cells were gated and expression of CCR7 and CD45RA was examined. Patient's almost all CD8+ cells are terminally differentiated effector memory (T_EMRA_) expressing CD45RA+/CCR7-.

CD8+ T cells have been further classified into naïve (T_N_), central memory (T_CM_), effector memory (T_EM_), and terminally differentiated effector memory (T_EMRA_), and have been characterized extensively for phenotype and functions [16–19]. Therefore, we examined the characteristics of CD8 T cells in peripheral blood with multicolor flow cytometry. More than 80% of CD8+ T cells were LGL. Majority of CD8+ T cells (96%) in the patient were CD8+CCR7-CD45RA+ T_EMRA_ cells, whereas T_N_ (CD8+CCR7+CD45RA+), T_CM_ (CD8+CCR7+CD45RA-), and T_EM_ (CD8+CCR7-CD45RA-) were markedly reduced (Figure 2).

**Figure 3:**
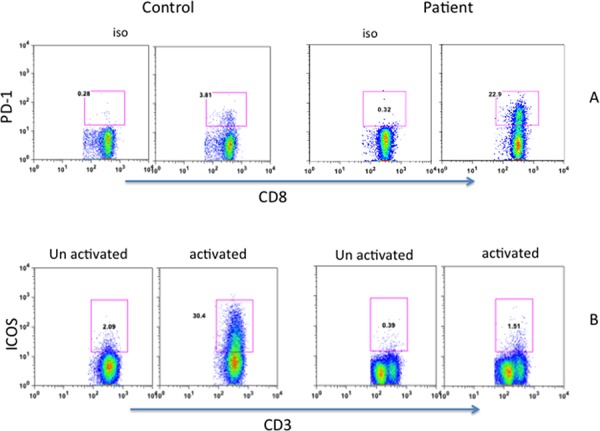
Expression of PD-1 A. and ICOS B PD-1 expression was observed on fresh cells, whereas ICOS expression was examined on 48 hours activated T cells. Increased PD-1 and decreased ICOS expression were observed in patients cells.

PD-1 (CD279) is one of the inhibitory molecules that are expressed on cells of the immune system, including peripheral CD8+ T cells, and persistently high expression of CD279 indicates an exhausted and dysfunctional T-cell phenotype [20–22]. Therefore, we examined the expression of CD279 on CD8+ T cells. Increased proportions of patient's CD8+ T cells expressed CD279 (22.9%) as compared to control (3.8%) (Figure 3A). The inducible co-stimulatory molecule (ICOS or CD278) is a member of CD28/CTLA4 family, which is expressed on activated T cells, and is essential for T cell activation [23–25]. Therefore, CD278 expression was examined on T cells activated with anti-CD3/CD28 after 48 hours (Figure 3B). A markedly reduced expression of CD278 was observed on patient's T cells (1.5% CD278+) as compared to control (30% CD278+).

**Figure 4:**
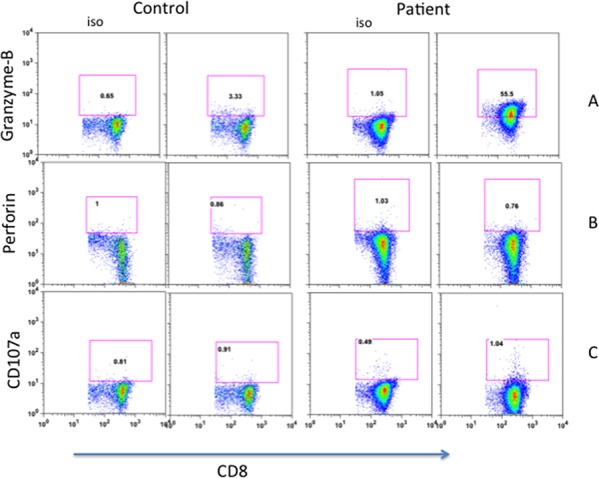
Expression of Granzyme-B A. perforin B. and CD107a C in CD8+ T cells. Increased expression of Granzyme in patients' CD8+ T cells.

One of the mechanisms of cyototoxic T lymphocyte-mediated cytotoxicity of target cells is by granzyme B, in a perforin-dependent and -independent manner. Therefore, we examined the expression of granzyme B and perforin of freshly isolated CD8+ T cells. Figure 4A show that significantly increased proportions of patient's CD8+ T cells expressed granzyme B (44.6%) as compared to CD8+ T cells from healthy control (2.5%); however, no significant different was observed in perforin expression between patient and control (Figure 4B). We also examined the expression of lsolysosomal-associated membrane protein-1 (CD107a), a marker of CD8+ T-cell degranulation following stimulation. No significant difference was observed in the expression of CD107a between freshly isolated CD8+ T cells from the patient and control (Figure 4C).

**Figure 5:**
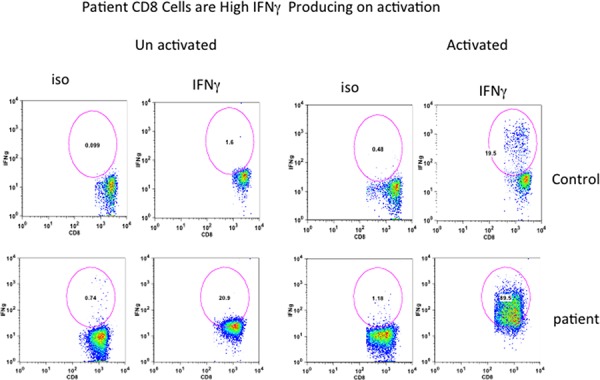
Intracellular IFN-γ in CD8+ T cells Cells were activated with PMA and Ionomycin and stained with anti-CD8 antibody, fixed, permeabilized, and stained with anti-IFN- antibodies and isotype controls. Almost all CD8+ T cells from patient are IFN-γ+.

Since CD8+ T cells secrete IFNγ (Tc1), we exa mined IFNγ producing CD8+ T cells from the patient and control. MNC were activated with PMA and ionomycin, and then incubated with beferidine to stop secretion of cytokines, and examined for intracellular IFNγ. Almost all CD8+ (98%) cells were positive for IFN-γ as compared to only 19% in healthy control (Figure 5).

**Figure 6:**
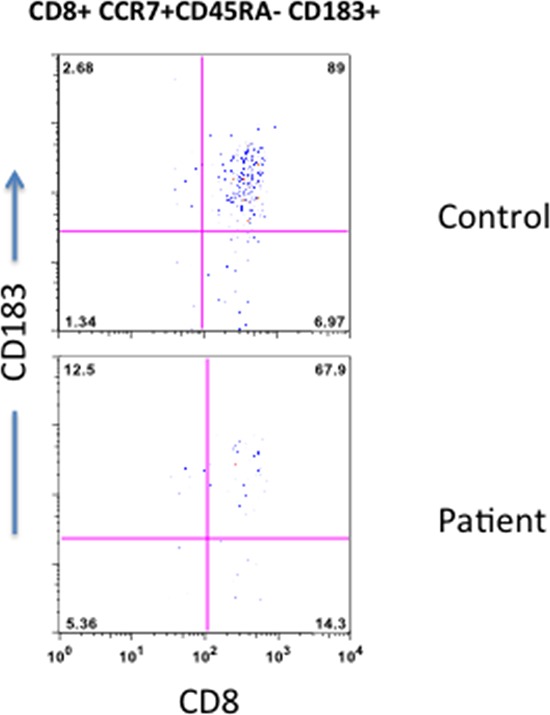
CD8 Treg cells Peripheral blood T cells were stain with anti-CD8, anti-CD183, anti-CCR7, and anti-CD45RA antibodies and isotypes. CD8+ T cells were gated and examined the expression of CD183, CCR7, and CD45RA. CD183+CCR7+CD45RA- CD8+Reg cells are reduced in the patient.

Recently interest in CD8 Treg has renewed. CD8 Treg suppress responses of both CD8+ and CD4+ T cells, and they have been shown to play a role in a number of autoimmune diseases [26–30]. CD8 Treg (CD8+CD183+CCR7+CD45RA-) were significantly reduced in the patient as compared to control (Figure 6), which may contribute to autoimmunity in T-LGL.

## DISCUSSION

Good syndrome is characterized by thymoma and combined T and B cell deficiency with high frequency of opportunistic infections, and frequently with concurrent autoimmune diseases [1–8]. The diagnosis of Good syndrome in our patient was based upon presence of thymoma, pan-hypoimmunoglobulinemia, absence of B cells, impaired specific antibody responses and T cell functional deficiency.

Malignancies in Good syndrome are extremely rare. Benign gammopathies of underminant significance (MGUS) of IgM and IgG isotypes have been reported in patients with Good syndrome [8, 9]. T cell lymphoblastic leukemia/lymphoma has been described in a patient with concurrent invasive thymoma found at autopsy [31]. However, no evidence of immunodeficiency was reported. Therefore, patient did not meet the criteria for a diagnosis of Good syndrome. To best of our knowledge, our patient is the first case of leukemia in Good syndrome. Diagnosis of CD8+ T-LGL was confirmed by flow cytometry and clonality by PCR. It is believed that dysregulated activation signaling and impaired Fas-induced dealth signaling complex (DISC) formation, which renders LGL CD8+ T cells resistant to Fas-mediated apoptosis, enable them to proliferate and contribute to pathogenesis of LGL [32].

Naive T cells upon exposure to an antigen undergo a clonal expansion of effector cells, which after clearing the antigen, undergo a phase of contraction when antigen-specific T cells undergo apoptosis, and then a small number of antigen-specific T cells stabilizes and retained as memory T cells [16–19]. These memory T cells differentially express adhesion molecules and chemokine receptors, which allow them to home in peripheral blood lymphoid tissues. Based upon the expression or lack of them, memory CD4+ and CD8+ T cells migrate to lymph nodes and spleen (central memory, T_CM_) or to extralymphoid tissue like lung and liver (effector memory; T_EM_). A small subpopulation of T_EM_ cells re-acquires CD45RA, and are termed as T_EMRA_ or terminally differentiated memory or exhausted T effector cells. T_EM_ and T_EMRA_ T cells T cells display poor proliferation, decreased telomere length, and are resistance to apoptosis [18]. These subpopulations are accumulated in human aging and contribute to T cell immunosenescence [19, 33]. Since our patient's LGL leukemia is CD8+ type and 68% of peripheral blood T cells are CD8+, we further characterized CD8+ T cells. Almost patient's all CD8+ cells (>96%) were T_EMRA_ suggesting that LGL cells are a clonal expansion of T_EMRA_. We have reported that T_EMRA_ CD8+ T cells display increased levels of anti-apoptotic molecules and are resistant to death-receptor and mitochondrial pathway-mediated apoptosis [19]. This would be consistent with the observations that T-LGL cells have impaired death-inducing signaling complex formation resulting in resistance to FAS-mediated apoptosis, resulting in their autonomous proliferation [32]. Furthermore, a large proportion of CD8+ T cells (approx. 45%) were positive for granzyme B; the expression of CD107a, a marker for degranulation of cytotoxic granules following cellular activation, and perforin expression was comparable to control. Granzyme B can be seen in the bone marrow and splenic sinuses, and malignant cells display similar features of activated effector and cytotoxic T cells, such as perforin and granzyme [34].

A large proportions of patient's CD8+ T cells expressed CD279. CD279 is one of the inhibitory molecules that are expressed on cells of the immune system, including peripheral CD4+, CD8+ T cells, upon their activation [20, 21]. In the presence of chronic viral infection, CD8 T cells develop a transient dysfunctional or exhausted T-cell phenotype characterized by a higher expression of CD279 compared to functional T cells; however, persistently high expression indicates an exhausted and dysfunctional T-cell phenotype [20–22]. Therefore, T_EMRA_ phenotype along with increased CD279 expression suggests that patients' LGL CD8+ T cells are of exhausted phenotype.

The inducible co-stimulatory molecule (ICOS, CD278) is a member of CD28/CTLA4 family, which is expressed on activated T cells [23, 24]. CD278 is essential for T cell activation, proliferation, secretion of cytokines, and immunoglobulin isotype switch (23–25, 35). We have reported that T_EMRA_ CD8+ cells express only minimal amounts of CD278 [18]. CD278 expression was markedly decreased in patient's CD8+ T cells as compared to control CD8+ T cells. Therefore, accumulation of CD278^low^ CD8+ phenotype may contribute to T cell deficiency.

Granzyme B mediates its cytotoxic effects against target cells by in a perforin dependent and independent manner [36]. It remains to be determined whether increased granzyme B in CD8+ T cells may be responsible for killing of autologous lymphocyte CD4+ T cells and/or B cells, therefore, contributing further to CD4 and B cell lymphopenia, which is feature of Good syndrome without leukemia. Furthermore, an accumulation of CD279^high^CD278^low^ CD8 T_EMRA_ cells that display impaired activation and proliferation may also appears to contribute to severe T cell function deficiency. It remains to be determined whether non-monoclonal CD8+ T cells in Good syndrome also express similar phenotype.

More recently, CD8 Treg cells have been reported to play an important role in immune homeostasis [26, 27]. In our patient, CD8+ Treg cells (CD8+CCR7+CD183+CD45RA-) were reduced. This would be consistent with reduced CD8 T_CM_ cells, since CD8 Treg belongs to T_CM_ CD8+ population [26]. A role of CD8 Treg has been demonstrated in a number of animal models and autoimmune diseases in humans [28–30]. Therefore, a deficiency of CD8 Treg may play a role in high incidence or autoimmune phenomenon and autoimmune diseases in T-LGL, and neutropenia in our patient.

In summary, T-LGL associated with Good syndrome is a clonal expansion of CD279+granzyme+IFN-γ+ CD8+ T_EMRA_ cells.

## MATERIALS AND METHODS

Peripheral blood mononuclear cells (MNCs) were isolated from blood of patient and healthy subjects by Ficoll-hypaque density gradient. Protocol was approved by Human Subject Committee of the Institution Review Board, University of California, Irvine,

### Antibodies and reagents

The following monoclonal anti-human antibodies were used: CD8 PerCP, CD45RA APC, CCR7 FITC, CD183 PE, CD3 PerCP, CD278 (ICOS) PE, CD279 (PD-1) PE. All antibodies were purchased from BD Parmingen (San Jose, California).

### Immunophenotyping of T, T cell subsets, B cells, NK cells and memory subsets of CD8+ T cells

Peripheral blood mononuclear cells were analyzed for T cells (CD3+), T helper (CD3+CD4+), T cytotoxic (CD3+CD8+), NK (CD3-CD56+CD16+), B cells (CD19+), and naïve (T_N_), central memory (T_CM_), effector memory (T_CM_), and terminally differentiated effector memory (T_EMRA_) exhausted subsets of CD8+ T cells with monoclonal antibodies against CD3, CD4, CD8, CD56, CD16, CD19, CCR7, and CD45RA, and isotype controls using multicolor flow cytometry with FACSCalibur. Peripheral blood CD8+ T cells were further characterized for the expression of PD-1 (CD279) and ICOS (CD278). Cells were stained with antibodies as above panel for 30 min at 4°C, washed by phosphate buffered saline and Flow cytometry was performed using FACScalibur (Becton-Dickenson, San Jose, CA) equipped with argon ion laser emitting at 488 nm (for FITC, PE and PerCP excitation) and a spatially separate diode laser emitting at 631 nm (for APC excitation). Forward and side scatters were used to gate and exclude cellular debris. Ten thousand cells were acquired and analyzed using Flowjo software (Treestar, Ashland, OR). Since CD278 is expressed on activated T cells, ICOS expression was examined on T cells activated with CD3/28 Beads (Invitogen, San Diego) for 48 hours.

### Immunophenotyping of CD8 Treg

CD8 Treg were analyzed for phenotypic expression of CD8, CD183, CD45RA, and CCR7. Cells were stained with specific antibodies against above surface antigens and isotype controls. Ten thousand cells were acquired, multicolor flow cytometry was performed using FACSCalibur, and analyzed using Flowjo software. CD8 Treg were identified as CD8+CD183+CCR7+ CD45RA-.

### Detection of intracellular IFNγ

2 × 10^6^/mL Cells in RPMI160 medium were activated with10 ng/ml phorbol 12-myristate 13-acetate (PMA) + ionomycin 1 μg/ml and 10 μg/ml Brefeldin A (BFA) (from Sigma Aldrich, St. Louis, MO) Incubate for 4 hours at 37°C, 5% CO2. Cells were surface stained with anti-CD8 PerCP for 30 min at 4°C. Cells were fixed with 250 μl of BD Cytofix/Cytoperm™ Buffer. Cells were washed with BD Perm/Wash™ buffer. Unactivated and activated cells were stained for Intracellular IFNγ FITC and isotype control. Cells were acquired with FACSCalibur and analyzed by Flowjo software.

### Detection of granzyme-B, perforin, and CD107a

Cells were surface stained for CD8 PerCP and CD107a PE for 30 min at 4°C. Cells were fixed and permeabilized as by protocol by BD Cytofix/Cytoperm™ Buffer and BD Perm/Wash™ buffer. Cells were stained for intracellular granzyme B (Alexa647) and perforin (FITC), and isotype were used as background controls. Cells were analyzed by FACS Calibur and analyzed by Flowjo software.
